# Coronary Vascular (DYS) Function and Invasive Physiology Assessment: Insights into Bolus and Continuous Thermodilution Methods

**DOI:** 10.3390/jcm12144864

**Published:** 2023-07-24

**Authors:** Matteo Maurina, Alice Benedetti, Giulio Stefanini, Gianluigi Condorelli, Carlos Collet, Carlo Zivelonghi, Pieter C. Smits, Valeria Paradies

**Affiliations:** 1 Department of Biomedical Sciences, Humanitas University, 20072 Pieve Emanuele, MI, Italy; 2 Cardio Center, IRCCS Humanitas Research Hospital, 20089 Rozzano, MI, Italy; 3 Department of Cardiology, Maasstad Hospital, 3079 DZ Rotterdam, The Netherlands; 4 HartCentrum, Antwerpen Hospital Network (ZNA) Middelheim, 2020 Antwerp, Belgium; 5 Cardiovascular Center Aalst, OLV Clinic, 9300 Aalst, Belgium; 6 Department of Cardiology, Erasmus University Medical Center, Thoraxcenter, 3015 GD Rotterdam, The Netherlands

**Keywords:** ANOCA, INOCA, MINOCA, vasospastic angina, microvascular angina, coronary microvascular dysfunction, bolus thermodilution method, continuous thermodilution method

## Abstract

A considerable number of patients with angina or myocardial ischemia have no significant coronary artery disease on invasive angiography. In recent years, several steps towards a better comprehension of the pathophysiology of these conditions, angina or ischemia with non-obstructive coronary arteries (ANOCA/INOCA), have been made. Nevertheless, several gaps in knowledge still remain. This review is intended to provide a comprehensive overview of ANOCA and INOCA, with a particular focus on pathophysiology, recent diagnostic innovations, gaps in knowledge and treatment modalities.

## 1. Introduction

A considerable number of patients with anginal symptoms or myocardial ischemia on noninvasive testing present no significant coronary artery stenosis on coronary angiography [1]. The absence of significant coronary artery disease (CAD) often terminates the diagnostic workup for cardiac causes. In some cases, symptoms are attributed to non-cardiac conditions such as gastrointestinal or psychiatric disorders, leading to erroneous diagnoses and inappropriate treatments [2]. In recent years, the pathophysiology of angina or ischemia with non-obstructive coronary arteries (ANOCA/INOCA) have been investigated, and several steps have been made towards a better comprehension of these conditions. Nevertheless, several gaps in knowledge still remain. In this review, we aim to provide an overview of ANOCA and INOCA, with a special focus on pathogenesis, recent innovations in diagnostic modalities, evolving concepts and treatment updates.

## 2. Definitions

The concomitant presence of typical angina and absence of significant stenosis on coronary angiography has been historically defined as “Cardiac Syndrome X” (CSX) due to uncertainty about its mechanism [3]. The better understanding of this condition led to the replacement of the term “CSX” with microvascular angina (MVA) [4], which encompasses patients with coronary vasomotor dysfunction consisting of microvascular spasm and/or impaired vasodilatation [5,6]. While microvascular spasm can be inferred in patients with typical angina and ischemic electrocardiogram (ECG) changes during a spasm provocative test [7], impaired vasodilator reserve due to coronary microvascular dysfunction (CMD) is characterized by a low coronary flow reserve (CFR) [5]. Differently, a transient reduction in coronary blood flow (CBF) secondary to a spasm in the epicardial coronary arteries, previously referred to as “Prinzmetal” or “variant” angina, defines the condition of vasospastic angina (VSA) [7,8,9,10]. The pathophysiological mechanisms of these conditions are addressed later in this review.

**Figure 1:**
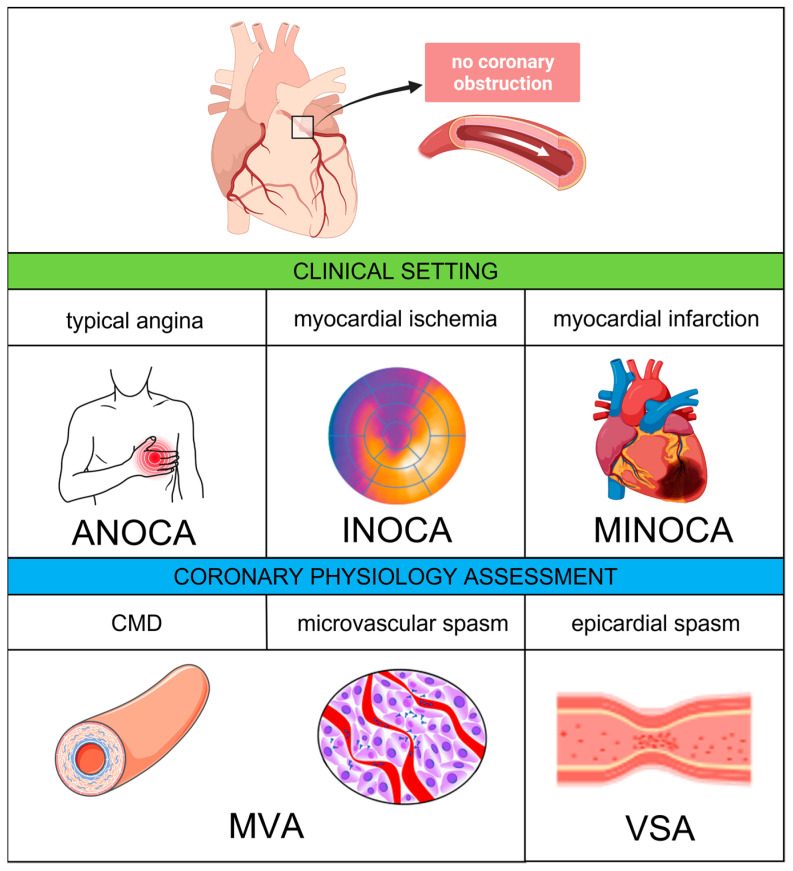
Coronary vascular dysfunction—clinical settings and pathophysiological mechanisms. Abbreviations: ANOCA, angina with non-obstructive coronary arteries; CMD, coronary microvascular dysfunction: INOCA, ischemia with non-obstructive coronary arteries; MINOCA, myocardial with non-obstructive coronary arteries; MVA, microvascular angina; VSA, vasospastic angina.

Recently, the term “ANOCA” has been coined to include all the clinical conditions determined by anginal symptoms without evidence of significant CAD on coronary angiography. When anginal symptoms are associated with documented myocardial ischemia, the term “INOCA” applies. Finally, in the presence of a universal criteria-defined myocardial infarction (MI) with no significant coronary stenosis and no other overt cause at presentation, the definition of “myocardial infarction with non-obstructive coronary artery” (MINOCA) can be used [11]. A comprehensive diagram including the abovementioned definitions is provided by Figure 1.

## 3. Epidemiology and Prognostic Implications

The absence of significant coronary obstruction is frequent in patients experiencing angina and myocardial ischemia. With regard to ANOCA, numerous data show that its prevalence ranges from 30% to 50% among patients undergoing coronary angiography [1,12,13,14], with a female preponderance [14,15,16]. Interestingly, these percentages seem to remain stable over time despite improvements in the definition and detection of this condition [12]. Nevertheless, the correct characterization of symptoms has a central role, since the presence of typical angina is a predictor of significant CAD, while atypical symptoms are more often associated with the absence of coronary obstruction [1,13]. When a non-invasive stress test confirms the presence of significant myocardial ischemia in patients with angina, the percentage of patients without significant CAD decreases [1,17]. The prevalence of MINOCA has been reported to range between 2% and 10% of all Mis [18,19,20,21,22], while coronary obstruction is found in more than 90% [23]. Interestingly, women have a higher chance of a negative coronary angiography than men also in the setting of MI [18].

Notably, none of these conditions is benign. In addition to impaired quality of life, several studies reported an increased risk of adverse events in patients with ANOCA and INOCA [24,25,26]. The available data show a 1.3 higher risk of death and a 3–4-fold higher risk of hospitalization for cardiovascular events in ANOCA patients as compared with asymptomatic individuals [14,25], while the Women’s Ischemia Syndrome Evaluation (WISE) study demonstrated a 10-year risk of all-cause death of 13% and 2.8% in patients with INOCA and in control healthy individuals, respectively [27]. In MINOCA patients, the prognosis is influenced by the underlying mechanism and the extent of myocardial dysfunction. Overall, the risk of mortality in patients with MINOCA is estimated to be about 1%, 3% and 4% in hospital, at 1 year and at 3 years, respectively [22,28,29]. Finally, the absence of significant coronary obstruction on coronary angiography may lead the treating physician to wrongly reassure patients or discontinue the medical therapy, with a potential negative impact on symptoms and quality of life.

## 4. Pathophysiology

Among the main pathophysiological mechanisms deemed responsible for ANOCA/INOCA and MINOCA, vascular spasm and CMD play an important role.

### 4.1. Epicardial and Microvascular Vasospasm

Epicardial vasospasm is a pathologic condition characterized by a transient reduction of CBF due to a spasm in the epicardial coronary arteries. This phenomenon is responsible for recurrent attacks of chest pain similar to those occurring in patients with obstructive CAD; however, these episodes often happen at rest rather than under exertion, and effort tolerance is generally preserved [30]. While initially referred to as “Prinzmetal” or “variant” angina due to its differences from classical effort angina, the exact definition of the pathophysiology of this phenomenon led to the introduction of the term “VSA”.

The hyperreactivity of vascular smooth cells (VSCs) is thought to play a central role in the pathophysiology of VSA. VSCs’ contraction is mediated by the phosphorylation and dephosphorylation of myosin light chains (MLCs). MLC dephosphorylation, in particular, is inhibited by rho-kinase, which has been shown to be hyper-expressed in the spastic cells [31,32]. This hypothesis is supported by preliminary studies suggesting that the use of rho-kinase inhibitors effectively prevents acetylcholine (Ach)-induced coronary spasms [33]. The occurrence of anginal symptoms at night, when the vagal tone is higher, and the induction of spasm by Ach suggest that dysregulation in the autonomic nervous system may be involved as well in the pathophysiology of VSA [8,34]. Other potential mechanisms include endothelial dysfunction with dysregulation of the nitric oxide synthases, magnesium deficiency, oxidative stress, inflammation and genetic polymorphisms [8]. Coronary spasm often occurs at the level of an atherosclerotic plaque, probably because the endothelial dysfunction, leading to an imbalance between vasodilator and vasopressor stimuli [35], can act as a trigger for local injury, ischemic damage and MI [36]. However, even healthy vessels can be affected.

Several studies suggest that a spasm can also occur at the level of the microcirculation. Mohri et al. showed that lactate was produced during a spasm provocation test in patients with angina attack and ischemic ECG changes without significant epicardial coronary obstruction [37]. Sun et al. confirmed these findings demonstrating the occurrence of myocardial ischemia (typical angina, ischemic ECG changes and lactate production) in patients undergoing the spasm provocative test without angiographic evidence of an epicardial spasm [38]. These data support the hypothesis that, in the absence of an epicardial spasm, patients with chest pain and ECG changes during the spasm provocative test suffer from microvascular spasm. Of note, it should be considered that patients with non-diagnostic Ach-test results may be affected by CMD [7]. Finally, one of the main characteristics of both epicardial and microvascular vasospasms is the resolution of symptoms due to vasodilation in response to nitrates.

### 4.2. Coronary Microvascular Dysfunction

CMD is a condition that is characterized by reduced CFR either due to increased minimal microvascular resistance (MVR) or high resting flow due to the altered non-endothelial-dependent vasomotion of the coronary microvasculature (<500 μm) [39,40,41,42]. Based on the underlying mechanism, two clinical entities can be distinguished, structural and functional CMD. The main structural changes associated with CMD are the thickening of the arterioles’ wall with a reduction in the lumen area, the increase in perivascular fibrous tissue and the decrease in the vascular density (capillary rarefaction), leading to high MVR. Additionally, non-endothelial-dependent mechanisms such as the impaired relaxation of VSCs, higher susceptibility of VSCs to vasoconstrictor stimuli and abnormal autonomic activity may be involved [43,44]. These abnormalities lead to an increase in the MVR and an insufficient increase in CBF under physiological stress, contributing to an imbalance between oxygen demand and supply, resulting in myocardial ischemia [5,41].

The parameter that expresses the ability of the coronary microcirculation to increase CBF under stress conditions is the CFR, defined as the ratio of the CBF during maximal vasodilation to the corresponding value at rest. Unlike other parameters used to assess the hemodynamic significance of a coronary stenosis, such as fractional flow reserve (FFR) and instantaneous wave-free ratio (iFR), CFR provides complementary information about the overall functional status of the coronary circulation. There can be a correlation between FFR, iFR and CFR in certain cases; for example, if a coronary stenosis is severe enough to limit CBF, then FFR, iFR and CFR may all be found to be altered. By contrast, other conditions, such as the presence of collateral circulation or CMD, may alter this relationship. In the case of well-developed collaterals supplying adequate CBF to the affected territory, CFR may remain preserved even in the case of an FFR and/or iFR significant stenosis. Furthermore, an altered CFR may suggest CMD in symptomatic patients with non-hemodynamically significant coronary stenosis at FFR and/or iFR assessment [45,46].

Typically, patients with CMD due to altered microvascular architecture present with low CFR and high MVR values. However, a proportion of patients with functional CMD may exhibit low CFR/low MVR and high resting CBF values. The mechanism responsible for this specific subset of CMD is still unclear, as this might be due to either a reduced myocardial efficiency or uncoupled CBF [47]. Structural changes in the coronary microvascular have been shown to be more prevalent in patients with classical cardiovascular risk factors, including hypertension, hyperlipidemia, smoking and diabetes mellitus (DM). Moreover, they have been correlated with other conditions, including renal impairment, coronary atherosclerosis, ventricular hypertrophy, and other cardiomyopathies [5,48].

While different findings on the coronary function tests (CFTs) may suggest that coronary microvascular spasm and CMD might represent two separated entities, an anomalous vasodilatory response has been proven in patients presenting the abovementioned risk factors and predisposition for structural abnormalities [48], suggesting that both non-endothelial-dependent and endothelial-dependent alterations may coexist in patients with MVA. Importantly, the coexistence of CMD and VSA portends a worse prognosis.

## 5. Diagnosis

Both non-invasive and invasive tests have a role in assessing coronary vascular function. While the discussion regarding non-invasive diagnostic tests is beyond the scope of this review, in the following paragraphs, the main invasive CFTs are discussed.

### 5.1. Spasm Provocation Test

The spasm provocation test is executed by injecting a spasm-inducing drug in escalating doses in the coronary arteries through a guiding catheter. Ach, which binds to the muscarinic cholinergic receptors, is the most widely used drug; however, ergonovine (ER) or methylergonovine can be employed too. There are few reports on the different responses to Ach and ER in patients with suspected VSA; however, it has been suggested that ER tends to provoke a more focal and proximal spasm, while Ach-induced spasm is more distal and diffuse [49].

Notably, Ach normally acts as a mild vasodilator on epicardial coronary arteries and dilates resistance arterioles, resulting in increases in CBF. In patients with normal endothelial function, blood flow increases >50% during low Ach doses. By contrast, the vasodilator response is attenuated at higher Ach doses in patients with VSA due to an imbalance between vasodilator and vasopressor agents, leading to vasoconstriction [50].

The spasm provocation test is performed by firstly engaging the target coronary artery. Of note, when performing the procedure via a radial artery access, the “radial cocktail” (namely Ca^2+^ channel blockers and nitrates) should be avoided. In the case of small radial arteries or marked spasm, 5F catheters are also considered a valid option to perform this assessment.

If there is no particular suspicion regarding a specific coronary segment involved in the spasm, the left coronary artery is normally the vessel of choice. During the entire duration of the test, a 12 leads ECG and the arterial blood pressure are continuously monitored. After a selective engagement of the left main, increasing doses of Ach are administered through the guiding catheter. Normally, four steps at the doses of 2, 20, 100 and 200 μg are sufficient to establish the diagnosis [51]. Ach is diluted in a 5, 10 or 20 mL syringe of 0.9% saline solution and is administered in 30–120 s based on the amount of solution used. A first cine acquisition is obtained before starting the injections; therefore, one is acquired after 20–30 s after each administration or in the case of new-onset angina or ECG changes. Intra-arterial nitrates should not be administered before starting the spasm provocation test, as they may alter the endothelial response, while they should be given at the end of the test. The test is safe, with an incidence of major complication < 1% [52]. In rare cases of acute spasm refractory to nitrates, atropine can be used. According to the COVADIS group [30], VSA is diagnosed if nitrate-responsive angina is accompanied by ECG changes and coronary spasm. Typical ECG changes include ST segment elevation or depression ≥ 0.1 mV or new-onset negative U waves, while the constriction in the epicardial arteries should be >90%. By contrast, the induction of typical angina and ECG changes in the absence of >90% epicardial spasm is highly suggestive of MVA due to a microvascular spasm.

**Figure 2:**
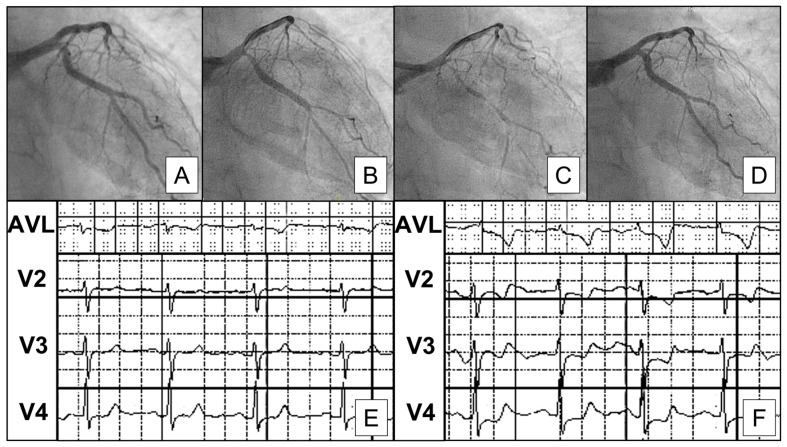
Spasm provocative test suggestive of vasospastic angina. (**A**) Baseline coronary angiography revealing no significant stenosis. (**B**,**C**) Repeated coronary angiography after administration of 2 μg and 20 μg of Ach showing significant spasm with >90% induced stenosis in the circumflex artery. (**D**) Repeated coronary angiography after administration of intracoronary nitrates revealing complete regression of coronary spasm. (**E**) Baseline electrocardiogram. (**F**) Electrocardiogram after administration of intracoronary Ach and during anginal symptoms, showing new-onset ischemic alterations in anterolateral position.

**Figure 3:**
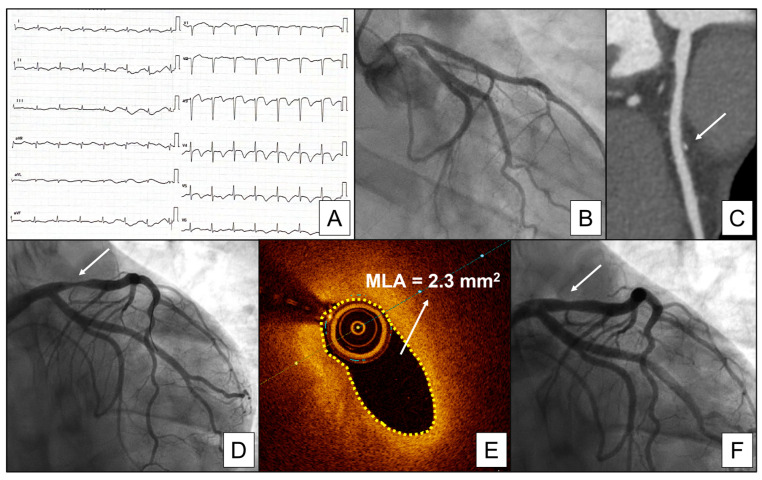
Representative case of coronary spasm on a mild coronary plaque in a patient presenting with out of hospital cardiac arrest. (**A**) Electrocardiogram at hospital presentation showing anterolateral ST segment elevation and biphasic T waves. (**B**) Coronary angiography revealing no significant stenosis. (**C**) Coronary computed tomography revealing a mild plaque with calcific core in the proximal LAD (white arrow). (**D**) Repeated coronary angiography showing significant spasm occurring on the proximal LAD at the passage of the OCT probe (white arrow). (**E**) OCT image showing coronary spasm. (**F**) Coronary angiography after administration of intracoronary nitrates showing the regression of the spasm (white arrow). Abbreviations: MLA, minimal lumen area.

Figure 2 provides an example of a positive vasospasm provocative test, while Figure 3 provides an exemplificative case of a coronary spasm that occurred at the level of a mild coronary plaque in a patient with resuscitated cardiac arrest.

### 5.2. Assessing CMD—General Concepts

In patients with suspected CMD, a comprehensive diagnostic test should include the measurement of specific indices reflecting the microvascular capacity to increase CBF under stress conditions and MVR. According to the COVADIS recommendations [7], these parameters include the CFR and the index of microvascular resistance (IMR). While CFR represents the extent to which CBF can increase above its baseline value, IMR is a dimensionless index that expresses the resistance of the microvasculature [53]. CFR reflects the status of both epicardial arteries and microcirculation, while IMR investigates the microvascular function [54]. Both of these parameters can be derived with the bolus thermodilution method; however, recent advances in the field of coronary physiology led to the development of a new diagnostic tool, the continuous thermodilution method, introducing new indices and overcoming some of the limitations of the bolus thermodilution method.

In addition to thermodilution, CFR and MVR can be estimated by applying the Doppler technique with a dedicated velocity-measuring wire [55]. Altered values of Doppler-derived CFR assessed with the Flowire (Philips Volcano, San Diego, CA, USA) have been correlated with markers of endothelial dysfunction, such as the albumin-to-creatinine ratio [56] and subendocardial viability ratio [57], in hypertensive patients. Several studies compared the thermodilution and the Doppler techniques in assessing CMD, leading to discordant results [58,59]. The Doppler method demonstrates lower intraobserver variability compared to thermodilution, leading to improved accuracy [59,60,61,62], and Doppler-derived CFR shows superior agreement with the gold-standard [15O]-H2O positron emission tomography [59,60]. Notably, the bolus thermodilution method tends to overestimate Doppler-derived CFR [63], while the continuous thermodilution and the Doppler method show an excellent agreement in assessing CFR [64]. However, the Doppler method’s primary drawback lies in its increased difficulty of execution and frequent constraints imposed by suboptimal signal quality [59,65]. Therefore, despite its technical limitations, the thermodilution is currently the most widely adopted method for CMD assessment.

### 5.3. Bolus Thermodilution Method

A dedicated guidewire with a pressure-temperature sensitive tip is used for both the bolus and the continuous thermodilution methods (PressureWire X, Abbott Vascular, Santa Clara, CA, USA). Notably, the bolus thermodilution method is based on the assumption that CBF equals the inverse of the transit time of blood and that the epicardial volume remains constant (i.e., Q ≈ 1/Tmn) [66].

**Figure 4:**
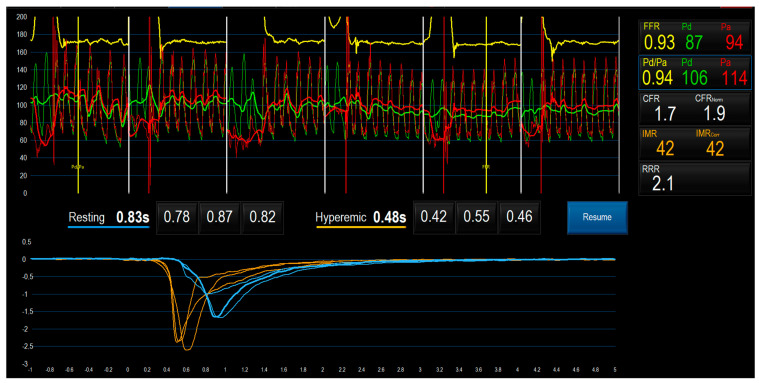
Microvascular assessment with the bolus thermodilution method showing high value of IMR (42 and low value of CFR (1.7), suggestive of coronary microvascular dysfunction.

First, the pressure sensor at the tip of the guidewire is equalized with the aortic pressure (Pa) just outside the guiding catheter, and then the wire is advanced in the coronary artery (normally to the distal left anterior descending coronary artery (LAD)). Subsequently, the temperature is zeroed to match the values in the two thermistors in the wire. The distal pressure (Pd), measured at the transition of the radiopaque part of the guidewire, and the Pa, measured at the tip of the fluid filled guiding catheter, are recorded simultaneously during the entire procedure. Then, the resting mean transit time (Tmn) is determined by the mean of three measurements obtained after three injections of 3 mL of saline solution in the coronary artery. To obtain reliable data, at least three injections should be performed, and transit-time values should not differ by more than 20% of each other. Operators should discard the measurements outside this range of reproducibility and continue with injections until three reliable transit time values with low variability are obtained. After determining the resting Tmn, the same procedure is repeated under hyperemia, which is generally achieved by continuous administration of adenosine through a central vein or intracoronary papaverine. A dose of adenosine 140 µg/kg/min or 12 mg of papaverine is sufficient to induce maximal hyperemia. CFR is calculated by dividing the resting Tmn by the hyperemic Tmn [67], while IMR is determined as the hyperemic Pd divided by the inverse of the hyperemic Tmn (IMR = Pd_hyper_ × Tmn_hyper_) [53,68], assuming that MVR is minimal during hyperemia (Table 1). The bolus thermodilution method further allows us to calculate the resistive reserve ratio (RRR), which is obtained by the ratio between the resting and the hyperemic MVR. This parameter is a marker of the microvasculature ability to change from baseline to minimal resistance during hyperemia [69]. According to the current recommendations, the cutoff values for normality are >2 for CFR and <25 for IMR [7,70]. Abnormal values of CFR (<2) and/or IMR (>25) are suggestive of CMD; nonetheless, given the overestimation of CFR by bolus thermodilution, a CFR gray zone of 2.0 to 2.5 has been proposed. With respect to the RRR, this parameter has been studied in patients with myocardial infarction, where a value ≤ 1.7 has been associated with an increased myocardial hemorrhagic area [71], but no specific cutoff has been established for CMD yet. A representative case of CMD assessment with the bolus thermodilution method is provided by Figure 4.

### 5.4. Continuous Thermodilution Method

Recently, the continuous thermodilution method was validated and introduced in many centers focused on coronary physiology. This method requires the use of a dedicated monorail infusion catheter (RayFlow, Hexacath Inc., Paris, France) composed by an inner lumen for the 0.014′′ pressure–temperature guidewire and an outer lumen for saline infusion [72]. After positioning the tip of the infusion catheter at the proximal target vessel (normally the LAD) and equalizing, the guidewire is advanced about 5–6 cm distal from the catheter tip. Then, the temperature is zeroed, and saline, at room temperature, is infused at prespecified flow rates. Generally, 10 and 20 mL/min saline infusion flow rates are chosen to assess coronary physiology parameters under resting and stress conditions, respectively. Intracoronary saline infusion at 20 mL/min induces hyperemia through both vasodilation and intravascular hemolysis and has been shown to be comparable to adenosine in assessing CFR [73]. After a temperature steady state is reached, the temperature of the mixture blood/saline (T) at the temperature sensor of the guidewire is measured for about 30 s. Thereafter, the guidewire is withdrawn into the infusion catheter to obtain the temperature of the infused saline (T_i_) [73,74]. As mentioned above, the procedure is repeated two times at different saline infusion flow rates to obtain measurements under both rest and stress conditions. Alternatively, the infusion can be programmed to automatically change from 10 mL to 20 mL and 10 mL, thus obtaining all the necessary values for the calculation of Q and R with one pullback.

**Figure 5:**
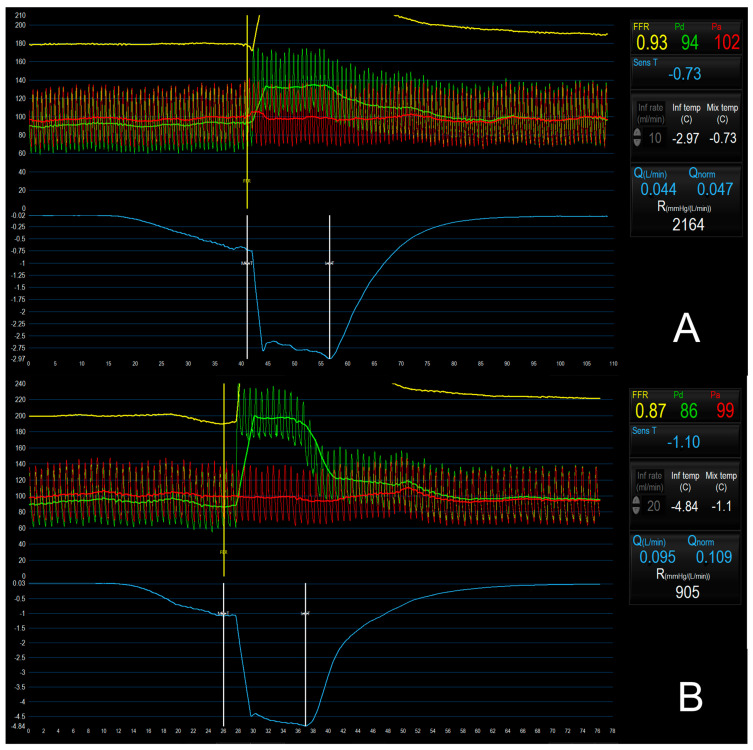
Microvascular assessment with the continuous thermodilution method. (**A**) Bolus thermodilution assessment during saline infusion reproducing resting conditions (10 mL/min). (**B**) Bolus thermodilution assessment during saline infusion reproducing hyperemia (20 mL/min), showing low values of Q (95 mL/min) and high value of R (905 UW) suggestive of coronary microvascular dysfunction.

The absolute coronary flow (Q) is calculated as the ratio of T_i_ to T, multiplied by the saline infusion rate (Q_i_) and corrected for a constant related to the difference between heat and density of blood and saline. The absolute microvascular resistance (R) is calculated as the ratio between the distal coronary pressure (Pd) and Q (Table 1) [74]. Notably, the repetition of these measurements under rest and stress conditions allows for the calculation of both CFR and of a novel index, the microvascular resistance reserve (MRR), which is obtained by the ratio between rest and hyperemic Q with a compensation for changes in blood pressure during hyperemic conditions and the presence of epicardial disease (Table 1). MRR is then the ratio of true resting microvascular resistance (Rµ, rest) as it would be in the hypothetical case that the epicardial artery would be completely normal, and hyperemic microvascular resistance (Rµ, hyp) expresses the capacity of the microvasculature to decrease its resistance under stress conditions [5,64]. Currently, the clinical application of Q, R and MRR is limited by the lack of validated cutoffs. However, recent studies have addressed the issue and proposed normality values. In a recent study assessing the relationship between CFR/IMR and Q and R, Konst et al. proposed a normality cutoff for Q and R, respectively, of 320 mL/min and 487 WU [62], while another study by de Vos et al. suggested that MRR values > 2.7 and <2.1, respectively, exclude and confirm the presence of CMD [75]. Despite the fact that its clinical application is still limited by its relatively recent introduction, the continuous thermodilution method has been validated against the gold-standard positron emission tomography (PET), showing a strong correlation and agreement between PET- and invasive-derived CFR [76]. In addition, it has proved to be safe and highly reproducible [74]. Figure 5 provides an exemplificatory case of CMD assessment with the continuous thermodilution method.

### 5.5. Bolus vs. Continuous Thermodilution Method to Assess CMD

Whether the bolus or the continuous thermodilution should be the method of choice in assessing CMD is a current matter of debate. Despite its recent introduction and the lack of validated cutoffs, the increasing uptake of the continuous thermodilution method in the catheterization laboratories opens a new window to a better understanding of microvascular dysfunction and to differentiate among different endotypes. Moreover, several aspects of this technique make it particularly interesting and potentially able to overcome some of the inherent limitations of the bolus thermodilution method. First, since adenosine is not required, assessing the microvascular function with the continuous thermodilution method represents a more tolerable procedure because unpleasant side effects, including tachycardia, chest oppression, and dyspnea, are eliminated. Second, adenosine is contraindicated in patients with bronchoconstrictive lung disease, asthma and second- or third-degree atrioventricular block, limiting the spectrum of patients eligible for the bolus thermodilution. Moreover, while the bolus thermodilution requires the physician to discard measurements with poor-quality curves or that are highly discordant, the continuous method is less operator dependent. Finally, the bolus thermodilution method is based on the assumption that CBF corresponds to the inverse of Tmn of the saline injection, while continuous-thermodilution-derived Q and R come from a direct measurement of true CBF [72]. Nonetheless, continuous thermodilution is more costly and requires an infusion pump that is able to be programed for the infusion of saline in mL/min.

In addition to the inherent diversities related to procedural factors, other aspects have been highlighted by recent studies comparing reproducibility, variability and symptoms prediction capacity of these two modalities. Gallinoro et al. showed that the variability of continuous-derived CFR and MRR is significantly smaller than of the bolus-derived CFR, IMR and RRR (12.8% vs. 31.3% for CFR_cont_ and CFR_bolus_; and 12.4% vs. 23.2% vs. 31.8% for MRR, IMR and RRR, respectively) [77]. Similar findings come from a recent study by Jansen et al. in which 73 patients underwent baseline and follow-up CFTs after 6 weeks. In this study, the authors showed that continuous-derived Q and R at 6 weeks significantly correlated with their baseline measurements, while bolus-derived CFR and IMR values did not [78]. The variability of bolus-derived CFR between baseline and follow-up might be explained by different resting flow values between the first and the second measurement. By contrast, the variability of the IMR values might be related to the intra-observer inconsistency of bolus transit times within different procedures.

With respect to their clinical relevance, de Vos et al. found that continuous-thermodilution-derived CFR and MRR were significantly associated with quality of life and angina domains, while the corresponding bolus-thermodilution-derived parameters were not. Notably, the correlation between symptoms and continuous-derived CFR and MRR further improved after excluding patients with spasm [79]. Another study by Konst et al. similarly showed that high values of continuous-thermodilution-derived R and/or low Q were more frequent in patients with severe angina, while bolus-thermodilution-derived CFR and IMR did not correlate with symptoms [62]. These results suggest that continuous- rather than bolus-thermodilution-derived parameters relate better to anginal symptoms.

The available data seem to suggest that the continuous thermodilution represents a more reliable and operator-independent method to evaluate the microvascular function. However, more validation studies are needed before this becomes the reference method to assess CMD.

## 6. Treatment

The management of patients with ANOCA/INOCA should be based on multidisciplinary counselling evaluating lifestyle and risk factors. The main cardiovascular risk factors, such as obesity, hypertension, dyslipidemia, DM and smoking, may be involved in the pathophysiology of CMD and epicardial or microvascular spasm. Therefore, lifestyle changes and risk factors’ control are the first-line recommendations in this category of patients [70]. Additionally, it is well known that physical exercise is associated with improvement in the endothelial function through nitric oxide (NO) formation and endothelium-dependent vasodilation [80]. The anti-inflammatory properties of statins and their effects on the endothelial function might be beneficial in patients without significant CAD [81]. Moreover, previous studies evaluating the efficacy of statins on CMD showed a significant improvement of CFR values in patients treated with atorvastatin [82] or rosuvastatin [83,84]. Hypertension is often associated with CMD, and angiotensin-converting enzyme inhibitors have been proven to ameliorate exercise tolerance and angina symptoms in patients with MVA [85]. Notably, patients treated with long-term enalapril showed an improvement of coronary microvasculature function and myocardial ischemia due to an increased bioavailability of endothelial NO [86]. In addition, the WISE study showed an increase of CFR values in women with CMD treated with quinalapril [87]. The Coronary Microvascular Angina (CorMicA) trial first demonstrated an improvement of angina symptoms and quality of life at 6 months and 1 year in patients treated with stratified medical therapy guided by invasive CFTs [88,89]. To simplify, a proper diagnosis between different ANOCA endotypes allows for a specific and distinct treatment which has been proven to improve the clinical outcome [90].

### Therapy of ANOCA

Beta-blockers may reduce ischemia symptoms in patients with MVA by reducing myocardial oxygen consumption and represent the first-line therapy recommended by the European Society of Cardiology [91]. However, only a few data are available about the effect of beta-blockers on microvascular function. Atenolol and propranolol have been shown to reduce ischemic episodes in patients with MVA [92,93], while intracoronary nebivolol administration showed a significant improvement in CFR values due to its vasodilatory effect [94]. Calcium channel blockers (CCBs) may be considered second-line therapy if beta-blockers are not tolerated, or as first-line therapy in patients with response to Ach testing suggestive of microvascular spasm [91]. Moreover, dihydropyridine CCBs such as amlodipine may be added to beta-blockers if blood-pressure values permit it [90]. As far as it is known about non-dihydropyridine CCBs and microvascular function, diltiazem failed to improve CFR values in patients with MVA [95]. Data about the effect of nitrates on coronary microcirculation are limited, and long-acting nitrates seemed ineffective, poorly tolerated or even detrimental in patients with MVA [96,97]. Nicorandil is a potassium-channel activator with a coronary microvascular dilatory effect and may be considered antianginal therapy [90]. Ranolazine is an antianginal drug that improves myocardial perfusion; however, data about its impact on coronary microcirculation are scarce. A randomized placebo-controlled trial showed that ranolazine therapy did not improve symptoms or myocardial perfusion in patients with no-obstructive CAD and CMD but improved myocardial perfusion in a subgroup of patients with low CFR values [98]. Finally, ivabradine may reduce angina symptoms by decreasing heart rate at rest and during exercise. However, studies evaluating the effect of ivabradine on microvascular function showed conflicting results [99,100]. Importantly, patients with CMD due to low CFR/low MVR and high resting CBF are unlikely to benefit from vasodilating drugs, while it could be theoretically more appropriate to specifically act on myocardial efficiency or uncoupled CBF [47].

CCBs should be considered first-line therapy and are effective in treating the majority of patients with epicardial vasospasm [101]. In some patients, high-dose CCBs or even a combination of non-dihydropyridine and dihydropyridine CCBs, such as diltiazem and amlodipine, is needed [70,102]. Despite the fact that long-acting nitrates are not suggested in patients with MVA, they showed a good efficacy in the treatment of VSA [103] and may be added in some patients. On the contrary, beta blockers are not recommended, as they might favor spasm by reducing the beta-mediated vasodilation, which opposes the alpha-mediated vasoconstriction [104].

## 7. Gaps in Knowledge

In the last decades, there has been a growing interest in coronary physiology assessments for a proper diagnosis of ANOCA and INOCA patients. Many studies investigated the diagnostic accuracy of invasive CFTs and their impact on clinical outcomes. However, some aspects of these techniques are still a matter of debate.

Previous studies revealed a modest correlation between Doppler-derived and bolus-thermodilution-derived CFR and a trend towards CFR overestimation with the bolus thermodilution method [59,63]. For this reason, some authors suggested a CFR threshold of 2.5 when measured with the bolus thermodilution method for better diagnostic accuracy of CMD [63]. Accordingly, a recent study by Gallinoro et al. found higher values of CFR when derived from bolus thermodilution than from continuous thermodilution [77]. Thus, further investigations are needed to confirm the better accuracy of higher cutoff values for bolus-thermodilution-derived CFR for the diagnosis of CMD.

One of the main limitations of these tests is still the uncertainty regarding their prognostic implications. Previous data demonstrated that a low CFR is associated with the worst clinical outcomes [105,106,107], while the clinical impact of high MVR values is still unclear. In a recent study, Boerhout et al. showed that the risk of MACE was higher in patients with an abnormal CFR regardless of normal or abnormal MVR [108]. Therefore, further studies evaluating the long-term follow-up of patients affected by CMD will shed light on the clinical impact of augmented MVR. Moreover, further evidence is needed to clarify the clinical implications of an abnormal MRR index.

Due to its wide myocardial perfusion territory, the LAD is typically the target vessel in which coronary physiology measurements are performed. CFTs can be performed in the left circumflex or right coronary artery in the case of technical issues in testing the LAD, or in addition to the LAD assessment in the case of negative results but a high clinical suspicion of CMD [70]. However, solid data about the comparison between CFR and MVR values measured in different myocardial territories are not available. In addition, it is unclear whether INOCA patients with a defined regionality of myocardial ischemia might present different coronary physiology features between the ischemic and the non-ischemic territories.

As mentioned above, recent data suggested the superiority of the continuous thermodilution over the bolus thermodilution method in assessing the microvascular function, mainly due to its better reproducibility, precision, and correlation with symptoms. As a consequence, it has to be investigated whether the continuous thermodilution method would increase the diagnostic accuracy of different CMD endotypes, such as coronary microvascular function presenting an isolated increase of MVR with normal CFR on bolus thermodilution, and, therefore, it is necessary to identify those patients who may benefit from specific and individualized therapies. In this context, the Netherlands registry of invasive Coronary vasomotor Function Testing was initiated with the aim of collecting baseline, procedural and follow-up data in patients with suspected and/or established ANOCA and INOCA. This registry will provide more insight into the pathophysiology, diagnostic process, and treatment of these patients, with potential implications from both scientific and clinical perspectives [6].

## Figures and Tables

**Table 1:** Coronary-function-testing-derived physiological parameters.

Invasive Method	Parameter	Formula	Cutoff for CMD	Meaning
Bolus thermodilution	Coronary flow reserve (CFR_bolus_)	CFR_bolus_ $=\frac{resting\;Tmn}{hyperemic\;Tmn}$	<2.0 (validated)	The capacity to increase CBF from resting to stress conditions (derived from transit time).
	Index of microvascular resistance (IMR)	IMR = hyperemic Pd×hyperemic Tmn	≥25 (validated)	Dimensionless index which represents MVR during stress conditions.
	Resistive reserve ratio (RRR)	RRR = $\frac{resting\;Pd\;\times \;resting\;Tmn}{hyperemic\;Pd\;\times \;hyperemic\;Tmn}$	Unknown	The ability of microcirculation to reduce MVR during stress conditions (derived from transit time).
Continuous thermodilution	Absolute coronary flow (Q)	Q = $\frac{Ti}{T}\times Qi\times 1.08$	<200 mL/min (under investigation)	Direct measurement of CBF.
	Absolute microvascular resistance (R)	R = $\frac{Pd}{Q}$	>500 UW (under investigation)	True MVR derived from CBF.
	Coronary flow reserve (CFR_continuous_)	CFR_continuous_ = $\frac{hyperemic\;Q}{resting\;Q}$	<2.0–2.5 (under investigation)	The capacity to increase CBF from resting to stress conditions (derived from CBF).
	Microvascular resistance reserve (MRR)	$\mathrm{MRR}=\frac{CFR}{FFR}\;\times \;\frac{resting\;Pa}{hyperemic\;Pa}$	<2.1 (under investigation)	The ability of microcirculation to reduce MVR during stress conditions (derived from CBF).

## Data Availability

No new data were created or analyzed in this study. Data sharing is not applicable to this article.
